# Differential Transcriptome Responses in Human THP-1 Macrophages Following Exposure to T98G and LN-18 Human Glioblastoma Secretions: A Simplified Bioinformatics Approach to Understanding Patient-Glioma-Specific Effects on Tumor-Associated Macrophages

**DOI:** 10.3390/ijms24065115

**Published:** 2023-03-07

**Authors:** Micaela R. Scobie, Abdullah Abood, Charles D. Rice

**Affiliations:** 1 Durham Veterans Health Administration Medical Center, Durham, NC 27705, USA; 2 Department of Biological Sciences, Clemson University, Clemson, SC 29634, USA; 3 Department of Biochemistry and Molecular Genetics, School of Medicine, University of Virginia, Charlottesville, VA 22903, USA

**Keywords:** LN-18 glioma, T98G glioma, THP-1 macrophage, tumor microenvironment, transcriptomics

## Abstract

A common theme in glioma disease progression is robust infiltration of immune cells within the tumor microenvironment, resulting in a state of chronic inflammation. This disease state is characterized by an abundance of CD68^+^ microglia and CD163^+^ bone marrow-derived macrophages with the greater the percentage of CD163^+^ cells, the poorer the prognosis. These macrophages are “cold,” in that their phenotype is of an alternatively activated state (M0-M2-like) supporting tumor growth rather than being engaged with classically activated, pro-inflammatory, and anti-tumor activities, referred to as “hot”, or M1-like. Herein, we have developed an in vitro approach that uses two human glioma cell lines, T98G and LN-18, which exhibit a variety of differing mutations and characteristics, to demonstrate their disparate effects on differentiated THP-1 macrophages. We first developed an approach to differentiating THP-1 monocytes to macrophages with mixed transcriptomic phenotypes we regard as M0-like macrophages. We then found that supernatants from the two different glioma cell lines induced different gene expression profiles in THP-1 macrophages, suggesting that from patient to patient, gliomas may be considered as different diseases. This study suggests that in addition to standard glioma treatment modalities, transcriptome profiling of the effects of cultured glioma cells on a standard THP-1 macrophage in vitro model may lead to future druggable targets that aim to reprogram tumor-associated macrophages towards an anti-tumor phenotype.

## 1. Introduction

Primary brain cancer and other central nervous system (CNS) cancers account for an estimated 16,606 deaths per year in the US alone, averaged over 2014–2018 [1]. In adults, gliomas comprise 22% of all brain and CNS tumors, and up to 78% of malignant brain and CNS tumors. Glioblastoma multiforme (GBM) (WHO grade IV) is the most aggressive and lethal subset of gliomas [2] and is found mainly in the cerebral hemispheres. GBM can arise from astrocytes, but other cell types contribute as well, including neuronal and oligodendrocyte precursors [3,4,5,6,7], which may determine the origin and nature of glioma stem cells [8].

GBM is notoriously invasive with rapidly growing vasculature by de novo angiogenesis and interactions with brain vasculature [9,10]. Median survival with treatment is only about 15 months and fewer than 30% of patients survive 2 years [11]. Post-surgical treatments typically involve both radiotherapy and chemotherapy with temozolomide (TMZ), yet unfortunately, relapse and rapid tumor regrowth and progression are typical outcomes [12,13]. Efforts to target angiogenesis associated with the growing GBM using anti-vascular endothelial growth factor (VEGF) monoclonal antibody Bevacizumab has failed to prevent tumor growth, though it does help to reduce cranial edema [14,15,16]. Other treatments, including immune check point inhibitors such as anti-PD-1, aim to impair ligation of PD-L1 expressed on glioma cells [17] and inhibitors of signal transduction in gliomas [18]. Unfortunately, these and other approaches have not been successful on a large scale.

Regardless of the cellular subtypes comprising gliomas, a common theme in glioma disease progression is robust infiltration of immune cells within the tumor microenvironment, resembling a state of chronic inflammation, with CD68^+^ microglia and CD163^+^ bone marrow-derived macrophages being the most abundant immune cell types, and the greater the percentage of CD163^+^ cells, the poorer the prognosis [19,20]. Upon closer examination by several investigators, these macrophages are “cold”, in that their phenotype is of an alternatively activated state (M0 M2-like) supporting tumor growth rather than being engaged with classically activated, pro-inflammatory, and anti-tumor activities, referred to as “hot” (M1-like) [21].

While the significance of glioma-associated macrophage activation being either M1, M0, or M2 is still debatable and perhaps patient-specific [21,22], there is a clear difference in cellular physiology of tumor-associated macrophages and their impact on tumor growth [23]. In particular, glioma-associated macrophages (GAM) in situ are pro-tumor by secreting M0-M2-like growth factors such as VEGF, IL-10, and other factors at the periphery of the growing tumor, and more M1-like in the necrotic core region [24]. It is possible that GAMs may be in a state of being alternatively activated upon recruitment by tumor cells (e.g., TGF-β) that aid tumor growth at the leading edge, but are subsequently metabolically switched to an inflammatory state in the hypoxic necrotic center via HIF-1α signaling and become part of the necrotic mass [20,24]. 

Translating current knowledge of the glioma microenvironment to clinical applications is needed by both the patient and medical community. Just as the composition, location, and progression dynamics are different from one glioma to another in different patients, the same may be true of the interactions between tumor-infiltrating macrophages across these dynamics. Therefore, a personalized approach may be an alternative to standard clinical approaches post-surgery. In the study herein, we have developed an in vitro approach that uses two human glioma cell lines, T98G and LN-18, which exhibit a variety of differing mutations and characteristics [25,26,27,28] to demonstrate their differential effects on differentiated THP-1 macrophages.

THP-1 cells are a human leukemia monocyte/histiocyte initially collected from a 1-year-old male with acute monocytic leukemia. The THP-1 cell line can be differentiated to macrophage-like cells in the presence of phorbol-12-myristate-13-acetate (PMA), and further manipulated to display features of classical or alternatively activated macrophages. For example, after PMA-induced differentiation, the cells can be treated with lipopolysaccharide (LPS) + IFN-γ, or IL-4 + IL-13 to yield M1-like and M2-like macrophages that express typical markers of their perspective phenotypes [29,30]. However, the sources of cytokines and preparations of LPS, and the levels of PMA used for differentiation, may vary dramatically between various published studies.

Furthermore, PMA is highly lipophilic and mimics diacylglycerol in activating protein kinase C, and thus elevated levels may drive the newly differentiated macrophage to full activation. In our study, we differentiated THP-1 cells with very low levels of PMA (16 nM) for 48 h followed by extensive washing, followed by another 48 h of incubation period without PMA, and show by transcriptomics that the resultant macrophage phenotype is neither distinctly M1 nor M2. Using this model, we then demonstrate that supernatants from confluent T98G and LN-18 glioblastoma cell cultures differentially influence the transcriptome of differentiated THP-1 macrophages. Resultant pathway analysis suggests that the tumor microenvironment associated with these two different glioma cells would be quite different, further suggesting perhaps that the patients would have responded differently to therapies targeting macrophage physiology within the tumor microenvironment. Therefore, we propose personalized approaches to understanding the effects of isolated and cultured glioma cells on macrophages as a part of treatment modalities.

## 2. Results

### 2.1. THP-1 Cells Treated with PMA Show Differences in Morphology Compared to Untreated THP-1 Cells

The untreated THP-1 monocytes maintained their rounded appearance and did not adhere to the flask (Figure S1A). When THP-1 monocytes were treated with 16 nM PMA for 48 h, followed by a 48 h resting period, their morphology changed from round to stellate. When compared to untreated THP-1 monocytes from the same cell culture passage, PMA-differentiated THP-1 cells adhered to the culture surface and became flattened with irregular shape and various membrane projections (Figure S1B).

### 2.2. PMA-Differentiated THP-1 Cells Display Distinct Genotypic Profile Compared to Undifferentiated THP-1 Monocytes and Have Positively Correlated logCPM Values among Biological Replicates

**Figure 1:**
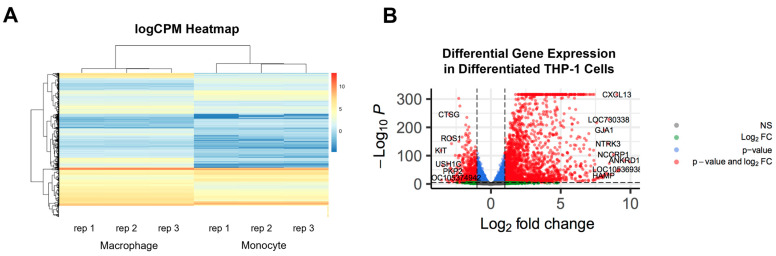
(**A**) Heatmap of log Counts Per Million (logCPM) of genes found in common between the THP-1 cells before and after differentiation (monocytes to macrophages). Replicates (rep) refer to biological replicates. (**B**) Volcano plot of differentially expressed genes (DEGs) in differentiated macrophages compared to monocytes. All genes shown as dots. Significantly DEGs (*p* < 0.05) are colored above the horizontal line. Genes with DGE > |1| are shown in red left and right of the vertical lines. Genes are only labeled with *p* < 0.001 and FDR < 0.001.

Heatmap clustering shows positively correlated logCPM values among THP-1 monocytes, and among PMA-differentiated cells, with a clear distinction between the two groups (Figure 1A). Via differential expression analysis of THP-1 monocytes and PMA-treated THP-1 cells, a total of 16,952 genes as high expression (logCPM > 1) were identified. Volcano plotting of these differential gene expression (DGE) data reveals a clear distinction between the expression profiles of THP-1 monocytes and PMA-treated THP-1 cells (Figure 1B). By applying an adjusted *p*-value < 0.01 and a fold change (FC) threshold of > |1|, a total of 3772 transcripts (2732 up-regulated and 1040 down-regulated) were found as differentially expressed genes (DEGs). Among the top 50 DEGs (based on highest logFC) are the matrix remodelers *MMP9*, *MMP12*, and *CHIT1*, the pro-fibrotic transcription co-regulator *ANKRD1* [31], and the M1 macrophage marker *CCR7*.

### 2.3. Key Genes Associated with Macrophage Differentiation and Mixed M1/M2-like Genotypes Are Up-Regulated in PMA-Treated THP-1 Cells

**Figure 2:**
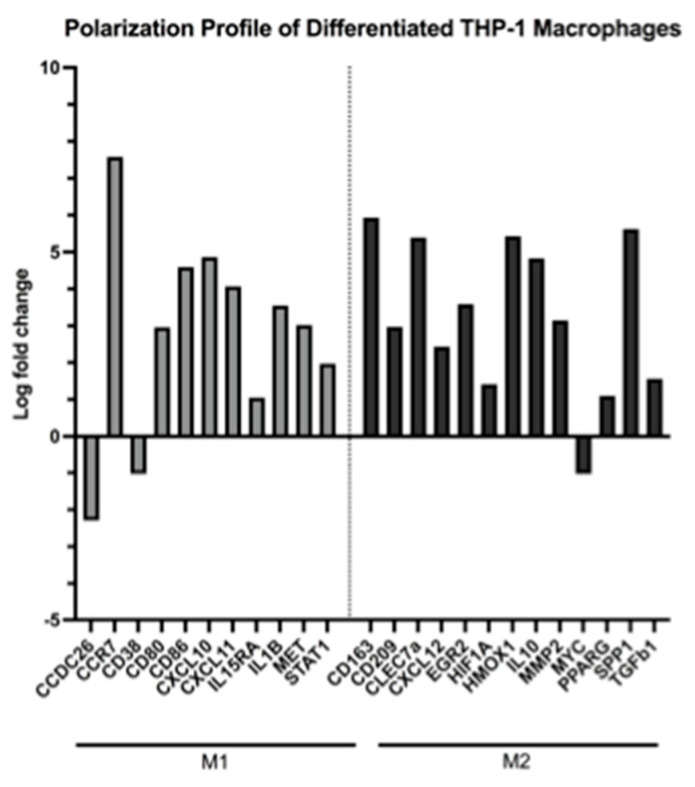
Histogram of select differentially expressed genes involved in M1 (**left**) or M2 (**right**) macrophage polarization according to the literature. Each bar represents the log fold change per gene as expressed in untreated macrophages compared to untreated monocytes. DEGs were included with *p* < 0.05 and FDR < 0.05.

Compared to THP-1 monocytes, PMA-differentiated THP-1 cells experienced significantly up-regulated gene expression of literature-backed macrophage differentiation markers. Specifically, KEGG pathway analysis [32] revealed pathway hsa04640 (Hematopoietic Cell Lineage) as significantly up-regulated. In addition, key genes involved in macrophage differentiation were differentially up-regulated, including *HLA-DRA*, *IL4R*, *CSF1*, *CSF1R*, *ITGAM*, and *ATP6VIF* (Table 1). Under the conditions of this study, differentiated THP-1 cells exhibited a macrophage-like population of mixed polarization genotype. DEGs associated with both M1-like and M2-like macrophages were up-regulated (Figure 2). Up-regulated genes associated with M1 polarization include *CCR7*, *CD80*, *CD86*, *CXCL10*, *CXCL11*, *IL15RA*, *IL1B*, *MET*, and *STAT1*. [22,33,34,35,36] Up-regulated genes associated with M2 polarization include *CD163*, *CD209*, *CLEC7A*, *CXCL12*, *EGR2*, *HIF1A*, *HMOX1*, *IL10*, *MMP2*, *PPAR*, *SPP1*, and *TGFB1*. [22,33,34,37,38,39,40,41,42].

### 2.4. Glioma LN-18 or T98G Secretions Show Distinct Impact on Gene Expression Profile of THP-1 Macrophages

**Figure 3:**
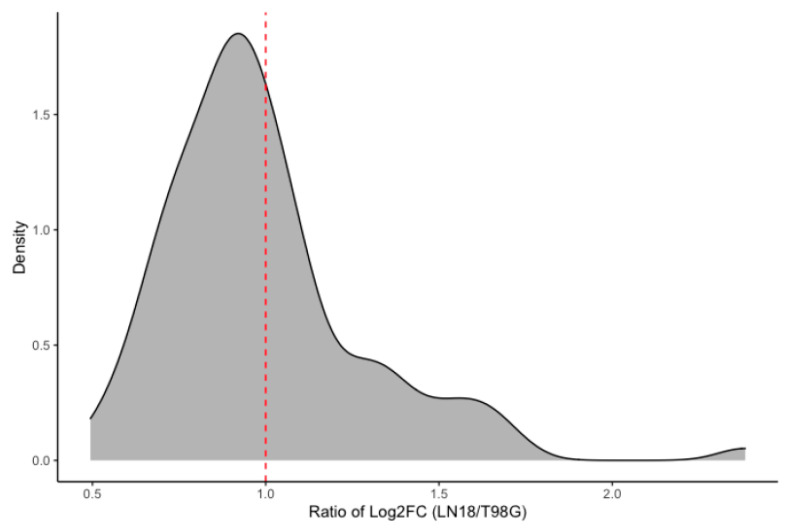
Ratio plot showing the intersection of genes that are differentially expressed in both macrophages treated with LN-18 supernatants and macrophages treated with T98G supernatants. Plotted on the *x*-axis is the ratio of log2FC in macrophages treated with LN-18 supernatants divided by the log2FC in macrophages treated with T98G supernatants. Plotted on the *y*-axis is the density (or number) of genes. Higher numbers of genes (density) falling below 1.0 indicate mutually expressed genes that are more highly differentially expressed in T98G-treated as compared to LN-18-treated THP-1 macrophages. Relatively lower densities falling above 1.0 indicate mutually expressed genes that are more highly DGE in LN-18-treated compared to T98G-treated THP-1 macrophages.

The effects of human glioblastoma secretions on the macrophage transcriptome reveal clear differences between LN-18 and T98G GBM cell lines. Among mutually expressed genes between the two treatment groups, a ratio plot (Figure 3) highlights the skewed distribution around 1.0, or when logFC_LN-18_ = logFC_T98G_. There is higher density of genes when logFC values of T98G are greater than the logFC values of LN-18 treated cells. This highlights that even though some of the genes are shared (differentially expressed in both treatment conditions), there are marked differences in the profiles of expression. Clearly, the macrophage gene expression profile is affected differently when treated with different glioma secretions.

**Figure 4:**
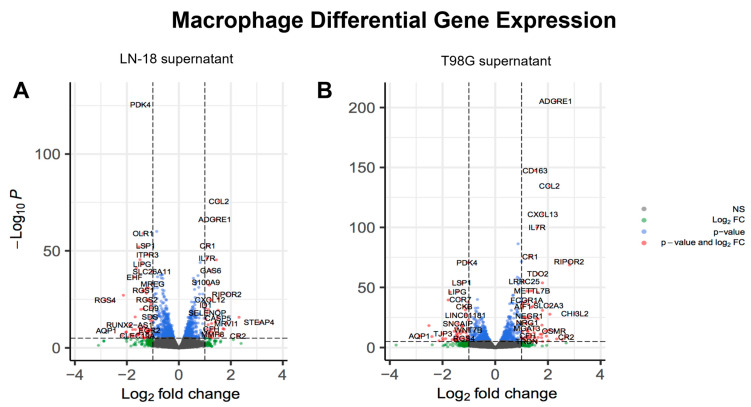
Volcano plot of differentially expressed genes in (**A**) macrophage treated with LN-18 control supernatant compared to untreated macrophage and (**B**) macrophage treated with T98G control supernatant compared to untreated macrophage. All genes shown as dots. Significantly differentially expressed genes (*p* < 0.05) are colored above the horizontal line as blue or red. Genes with DGE > |1| are shown in red left and right of the vertical lines and are all labeled.

**Figure 5:**
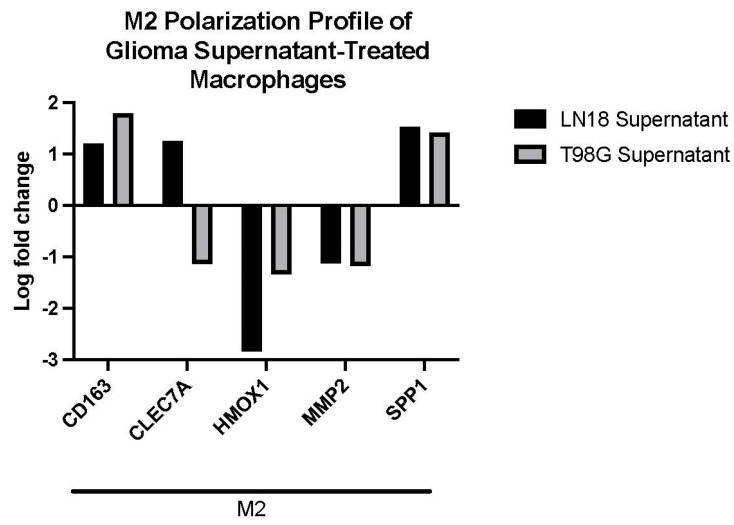
Histogram of differentially expressed genes involved in M2 macrophage polarization in either macrophages treated with supernatant exposed to LN-18 or T98G glioma cells. No M1 genes were found to be significantly differentially expressed.

Genes relevant to M1-like or M2-like macrophage genotype were selected post-DGE analysis for further consideration. LN-18 and T98G secretions showed similar effects on M2-like gene expression in THP-1 macrophages (Figure 4A,B). Both GBM cell lines caused up-regulation of *CD163* and *SPP1*, and down-regulation of *HMOX1* and *MMP2* in THP-1 macrophages. Gene expression of adhesion protein *CLEC7A* was up-regulated by exposure to LN-18 secretions, but down-regulated by T98G secretions (Figure 5). No selected M1-like genes, including *CCDC26*, *CCR7*, *CD38*, *CD80*, *CD86*, *CXCL10*, *CXCL11*, *IL15RA*, *IL1B*, *MET*, and *STAT1* were differentially expressed (not shown). A principal component analysis (PCA) plot for all samples sequenced and a heatmap of logFC of all genes between THP-1 macrophages treated with LN-18 or T98G supernatant can be found in supplementary Figures S2 and S3.

GO term enrichment using PANTHER [43] showed many significantly enriched GO terms from either up- or down-regulated gene sets (Table 2 and Table 3). Enriched Biological Process (BP) GO terms up-regulated (Table 2) in THP-1 macrophages exposed to either LN-18 or T98G secretions include Immune Complex Clearance and endogenous Lipid Antigen Processing and Presentation via MHC class Ib. Enriched GO terms uniquely up-regulated in THP-1 macrophages exposed to LN-18 secretions include Negative Regulation of Vascular Endothelial Cell Proliferation and Regulation of Astrocyte Activation. Enriched GO terms uniquely up-regulated in THP-1 macrophages exposed to T98G secretions include Negative Regulation of Glial Cell Apoptotic Process and Negative Regulation of IL-10 Production.

BP GO terms down-regulated (Table 3) in THP-1 macrophages exposed to either LN-18 or T98G secretions include Peptide Antigen Assembly with MHC Class II Protein Complex and MHC Class II Protein Complex Assembly. Enriched GO terms uniquely down-regulated in THP-1 macrophages exposed to LN-18 secretions include Antigen Processing and Presentation of Peptide Antigens. Enriched GO terms uniquely down-regulated in THP-1 macrophages exposed to T98G secretions include Defense Response to Gram-negative Bacterium and Cellular Amine Metabolic Process.

## 3. Discussion

In this study, we developed an approach to differentiating THP-1 monocytes to macrophages with mixed phenotypes we regard as M0-like macrophages. We also found that supernatants from different glioma cell lines induced different gene profiles in differentiated THP-1 macrophages. First, a comprehensive DGE analysis of human THP-1 cells differentiated with low levels of PMA over a 48 h period, followed by gentle washing and a 48 h period of resting, was carried out. Of note, the transcriptional profiles of PMA-treated THP-1 cells conformed to accepted macrophage genotype. These DGE data are now available to others in efforts to further refine and standardize the use of THP-1 macrophages.

Based on previous published studies, we chose a PMA concentration of 16 nM incubated for 48 h, followed by a 48 h recovery period. We hypothesized that this protocol would result in a fully differentiated macrophage, while reducing effect of any PMA-induced activation pathways. This was borne out by a mixed-polarization macrophage, with genes up- and down-regulated from both M1 and M2 profiles, indicating a neutral, or balanced, M0 starting phenotype and low activation. *CCDC26*, a long non-coding RNA gene, and *CD38*, a gene associated with activation and multiple myeloma, were suppressed, indicating that these cells were differentiated and no longer displaying the leukemia phenotype [44]. Expression of *MYC* was also suppressed in these cells, indicating that these cells were not actively going through cell cycling. Though not displayed herein, neither of the key genes associated with the unfolded protein response (*CHOP*, *GADD34*, and *XBP1*) were up- or down-regulated by the differentiation process, indicating that cells were not stressed. Furthermore, the PMA protocol used herein induced macrophage differentiation as determined by both morphology and gene expression profiles. Notably, gene sets with GO terms for macrophage activation, differentiation, and chemotaxis were significantly up-regulated. Macrophage “activation” is a loose and generalized term often referring to an increase in a wide variety of macrophage functions [45]. Throughout this paper, we use “macrophage activation” to indicate a macrophage that has been polarized to a differentiated state. Therefore, for this GO term to be significantly up-regulated implies only that PMA-treated THP-1 cells in our study are more macrophage-like in gene expression compared to the non-differentiated monocyte. 

In this study, we also found that supernatants from the two different glioma cell lines induced differential gene expression profiles in macrophages, though we have not identified the specific growth factor(s) responsible for such differences. T98G and LN-18 cells differ in their growth rate characteristics, in vitro invasiveness, morphology, TMZ resistance, *PDGF-Rα* expression, *VEGF-α* expression, PTEN/P13K profiles, and basal expression of the aryl hydrocarbon receptor (AHR) protein, as well as inducibility of AHR-associated *CYP1A1/CYP1B1* genes [26,27,28,46]. How these complex differences contribute to differential gene expression in supernatant-exposed macrophages should be the focus of future research.

Regardless of which individual or combination of growth factors are secreted by T98G and LN-18 cells, the clear difference in gene expression profiles suggests different signaling pathways were induced in macrophages expressing a combination of M0/M1 and M2 genes prior to treatment. Of note, genes typically associated with M1-like profile were not altered except for *CLEC7A*, also known as Dectin-1, a receptor for fungal-associated lectins, and this was observed in LN-18 cells, and not T98G. Macrophage dectin-1 expression has a role in anti-tumor activity by stimulating natural killer cell activity [47], leading to the speculation that LN-18 cells are susceptible to control by NK cells. Two genes typically associated with M2-polarizatioin, *CD163* and *SPPI*, were induced in macrophages treated with supernatants from both cell lines. Though limited in scope, these kinds of data give further in vitro support for the growing assumption that the glioma TME is polarized to an M2-environment that supports tumor growth. The observation that both *HMOX1*, which is associated with oxidative stress and hypoxia, and the metalloprotease *MMP2* were suppressed, suggests that macrophages with this overall expression profile may contribute to the transition of glioma cells from the more hypoxic and necrotic center to the normoxic leading edge of the growing tumor. This too should be the focus of future research.

Volcano plots of DGE in macrophages show a distinct difference between those treated with LN-18 vs. T98G supernatants. Upon examining DGE through the lens of biological responses from a list of GO terms (showing 10-fold enrichment) related to macrophage physiology, we found more up-regulated terms in LN-18 cells than T98G, and this was equally so for down-regulated genes. Taken as an overview, these GO terms, whether viewed as up- or down-regulation, indicate modulation of antigen processing and presentation, cellular metabolism, clearance of antigen–antibody complexes, and maintenance of an environment conducive to tumor growth. 

To date, any success in treatment for gliomas relies on slowing the time to recurrence following surgery, chemotherapy, and focused radiation. However, our study further supports the position that gliomas from different patients may be considered different diseases, with perhaps different outcomes if individualized intervention can be employed. We propose utilizing THP-1 macrophages differentiated with low levels of PMA for 48 h followed by a 48 h resting phase as a sentinel macrophage to then determine the immunomodulating effects of a patient’s cultured glioma cell supernatants at the transcription level. Though the common outcome may be polarization towards a pro-tumor M2 phenotype, analysis of DGE may lead to druggable targets that are individual patient-based. As an example, we previously demonstrated that indirubin E804, [Indirubin-3′-(2,3 dihydroxypropyl)-oximether] has anti-inflammatory properties in LN-18 and T98G glioma cells [46], which is significant because glioma cells are considered pro-inflammatory in nature by virtue of high levels of IL-6 secretion, but are also immunomodulatory via high levels of TGF-β secretion [48,49,50]. Future studies may reveal a means to target regulatory pathways in cytokine networks of the TME.

## 4. Materials and Methods

### 4.1. Cell Culture

LN-18 and T98G glioblastoma multiforme lines (ATCC CRL-2610; CRL-1690) and THP-1 monocytes (ATCC TIB-202) were maintained in T-75 flasks and cultured with Dulbecco’s Modified Eagle’s Medium (DMEM, Cellgro, MA, USA). DMEM was supplemented with 10% bovine fetal calf serum (FCS, Hyclone, GE Healthcare, Chicago, IL, USA), 1% non-essential amino acids (NEAA 100x, Lonza, Switzerland), 1% sodium bicarbonate, 1% Pen Strep Glutamine (P/S-G 100x, Gibco, Thermo Fisher Scientific, Waltham, MA, USA), 20 gentamycin sulfate (Thermo Fisher Scientific), and 4 μg/mL nyastatin (Thermo Fisher Scientific).

### 4.2. Treatments

Phorbol 12-myristate 13-acetate (PMA) (Sigma-Aldrich, St. Louis, MO, USA) was diluted in DMSO (Corning, New York, NY, USA) to a stock solution of 10^−2^ M and stored at −20 °C prior to use. THP-1 monocytes were seeded in twelve 6-well plates (Corning, New York, NY, USA) at 2 × 10^6^ cells per well in a final volume of 3 mL for a total design of four treatment groups with three replicates each. Cells in three of the four treatments were then differentiated using 16 nM (10 ng/mL) PMA for 48 h, washed extensively with fresh media, followed by a 48 h resting period in media only. Cells in the remaining treatment group served as an undifferentiated monocyte control by not receiving PMA during this period. Media from the monocyte control was removed and the cells were washed with cold PBS and dissolved in 1 mL TRIzol (Invitrogen, Waltham, MA, USA), and the contents stored at −80 °C prior to mRNA extraction. Cells in the remaining 9 plates were then covered with 1.5 mL media and 1.5 mL of supernatant from confluent T98G or LN-18 cultures, or media only as the macrophage control for 3 replicated experiments. After 24 h culture, all media was removed, cells were washed with cold PBS and dissolved in 1 mL TRIzol (Invitrogen, Waltham, MA, USA), and the contents stored at −8 °C prior to mRNA extraction.

### 4.3. Sample Preparation and mRNA Sequencing

Messenger RNA (mRNA) was extracted and purified by poly-a-tail enrichment. Strand-specific libraries were prepared by kit NEBNext^®^ Ultra™ II Directional RNA Library Prep Kit for Illumina^®^ (NEB E7760). Qualified libraries were then sequenced on an Illumina HiSeq 4000 Platform using a paired-end 150 run (2 × 150 bases). Sequencing depth was approximately 12 GB/sample. All samples were extracted and sequenced by Novogene Corporation, Inc. (Sacramento, CA, USA).

### 4.4. Data Analysis

Data quality control was performed using FastQC and trimmed for paired end data with Trimmomatic v-0.36. Reads were aligned to human reference genome GRCh38.p12 using GSNAP (Ensembl release 95 annotation), and mapped counts were summarized using Subread featureCounts (fragment length 50–600 base pairs). Raw counts were analyzed with edgeR to estimate the common negative binomial dispersion by conditional maximum likelihood (CML). Treatment effect was measured by calculating the log2 counts per million (CPM) of each gene and then using glm.ft to fit the generalized linear model. Unless otherwise noted, differential gene expression was considered statistically significant if both *p* values and the false discovery rate (FDR) were <0.05, with a log fold change (logFC) > |1|. For biologically relevant interpretation of differential gene expression profiles, enrichment of up- or down-regulated GO (Gene Ontology) terms was determined using PANTHER and significant KEGG (Kyoto Encyclopedia of Genes and Genomes) pathway enrichment using R/kegga.R.

## Figures and Tables

**Table 1:** Differential gene expression of genes involved in macrophage differentiation from the only significant KEGG pathway enrichment analysis (KEGG pathway hsa04640 “Hematopoetic Cell Lineage”). Genes included are major histocompatibility complex class II DR alpha (HLA-DRA), interleukin 4 receptor (IL4R), colony stimulating factor 1 (CSF1), colony stimulating factor 1 receptor (CSF1R), integrin alpha m (ITGAM), and ATPase H+ transporting v1 subunit F (ATP6V1F). Log FC = log fold change, Log CPM = log counts per million, LR = likelihood ratio, FDR = false discovery rate.

Symbol ID	Log FC	Log CPM	LR	*P*	FDR
HLA-DRA	3.01	2.79	258.12	4.40 × 10^−58^	4.60 × 10^−57^
IL4R (CD124)	1.23	5.72	386.22	5.52 × 10^−86^	8.90 × 10^−85^
CSF1 (M-SCF)	1.67	1.30	46.50	9.14 × 10^−12^	2.70 × 10^−11^
CSF1R (CD115)	1.87	8.19	1528.51	0	0
ITGAM (CD11b)	2.71	6.03	1762.36	0	0
ATP6V1F (CD14)	1.07	6.81	533.69	4.45 × 10^−118^	9.76 × 10^−117^

**Table 2:** Up-regulated GO enrichment analysis using the Panther database. Select enriched GO terms from up-regulated genes in THP-1 cells treated with LN-18 or T98G control supernatants. This is a subset of Biological Process (BP) terms from the full GO term list. GO terms are shown that had greater than 10-fold enrichment.

	Fold Enrichment	*P*	FDR	Ontology
**Up-regulated by LN18 supernatants**				
immune complex clearance (GO:0002434)	>100.00	9.40 × 10^−5^	1.84 × 10^−2^	BP
negative regulation of vascular endothelial cell proliferation (GO:1905563)	>100.00	1.97 × 10^−4^	3.24 × 10^−2^	BP
antigen processing and presentation, endogenous lipid antigen via MHC class Ib (GO:0048006)	>100.00	1.97 × 10^−4^	3.21 × 10^−2^	BP
antigen processing and presentation, exogenous lipid antigen via MHC class Ib (GO:0048007)	91.92	3.36 × 10^−4^	4.35 × 10^−2^	BP
regulation of astrocyte activation (GO:0061888)	>100.00	1.97 × 10^−4^	3.18 × 10^−2^	BP
leukocyte chemotaxis (GO:0030595)	19.70	1.23 × 10^−9^	6.40 × 10^−6^	BP
leukocyte migration (GO:0050900)	12.43	5.64 × 10^−8^	8.04 × 10^−5^	BP
negative regulation of dendritic cell apoptotic process (GO:2000669)	91.92	3.36 × 10^−4^	4.38 × 10^−2^	BP
positive regulation of microglial cell activation (GO:1903980)	80.43	4.19 × 10^−4^	4.97 × 10^−2^	BP
positive regulation of neuroinflammatory response (GO:0150078)	64.34	2.24 × 10^−5^	6.50 × 10^−3^	BP
positive regulation of inflammatory response (GO:0050729)	15.86	3.89 × 10^−7^	3.39 × 10^−4^	BP
**Up-regulated by T98G supernatants**				
immune complex clearance (GO:0002434)	>100.00	1.54 × 10^−4^	2.01 × 10^−2^	BP
antigen processing and presentation, endogenous lipid antigen via MHC class Ib (GO:0048006)	>100.00	3.22 × 10^−4^	3.63 × 10^−2^	BP
negative regulation of glial cell apoptotic process (GO:0034351)	83.70	1.29 × 10^−5^	3.06 × 10^−3^	BP
negative regulation of interleukin-10 production (GO:0032693)	39.65	8.77 × 10^−5^	1.26 × 10^−2^	BP

**Table 3:** Down-regulated GO enrichment analysis using the Panther database. Select enriched GO terms from down-regulated genes in THP-1 cells treated with LN-18 or T98G control supernatants. This is a subset of Biological Process (BP) terms from the full GO term list. GO terms are shown that had greater than 10-fold enrichment.

	Fold Enrichment	*P*	FDR	Ontology
**Down-regulated by LN-18 supernatants**				
peptide antigen assembly with MHC class II protein complex (GO:0002503)	50.96	2.44 × 10^−6^	2.55 × 10^−3^	BP
MHC class II protein complex assembly (GO:0002399)	50.96	2.44 × 10^−6^	2.39 × 10^−3^	BP
MHC protein complex assembly (GO:0002396)	40.77	5.27 × 10^−6^	3.59 × 10^−3^	BP
antigen processing and presentation of peptide antigen via MHC class II (GO:0002495)	25.48	2.79 × 10^−5^	1.07 × 10^−3^	BP
antigen processing and presentation of exogenous peptide antigen via MHC class II (GO:0019886)	27.18	2.22 × 10^−5^	9.93 × 10^−3^	BP
antigen processing and presentation of exogenous peptide antigen (GO:0002478)	14.83	1.98 × 10^−4^	4.09 × 10^−2^	BP
**Down-regulated by T98G supernatants**				
peptide antigen assembly with MHC class II protein complex (GO:0002503)	45.96	5.98 × 10^−5^	5.21 × 10^−2^	BP
MHC class II protein complex assembly (GO:0002399)	45.96	5.98 × 10^−5^	4.94 × 10^−2^	BP
defense response to Gram-negative bacterium (GO:0050829)	13.47	4.49 × 10^−5^	5.41 × 10^−2^	BP
cellular biogenic amine metabolic process (GO:0006576)	12.90	5.46 × 10^−5^	5.35 × 10^−2^	BP
cellular amine metabolic process (GO:0044106)	12.90	5.46 × 10^−5^	5.04 × 10^−2^	BP

## Data Availability

Sequencing data can be accessed at the GEO data repository (GSE226757).

## Supplementary Materials

The following supporting information can be downloaded at: https://www.mdpi.com/article/10.3390/ijms24065115/s1.
