# Supplementary material for: Differential Transcriptome Responses in Human THP-1 Macrophages Following Exposure to T98G and LN-18 Human Glioblastoma Secretions: A Simplified Bioinformatics Approach to Understanding Patient-Glioma-Specific Effects on Tumor-Associated Macrophages
Source: Int J Mol Sci. 2023 Mar 7;24(6):5115. doi: 10.3390/ijms24065115 (PMC10049238; doi:10.3390/ijms24065115)
Supplement: Supplementary file 1 [file ijms-24-05115-s001.zip › ijms-2254985-supplementary.pdf]

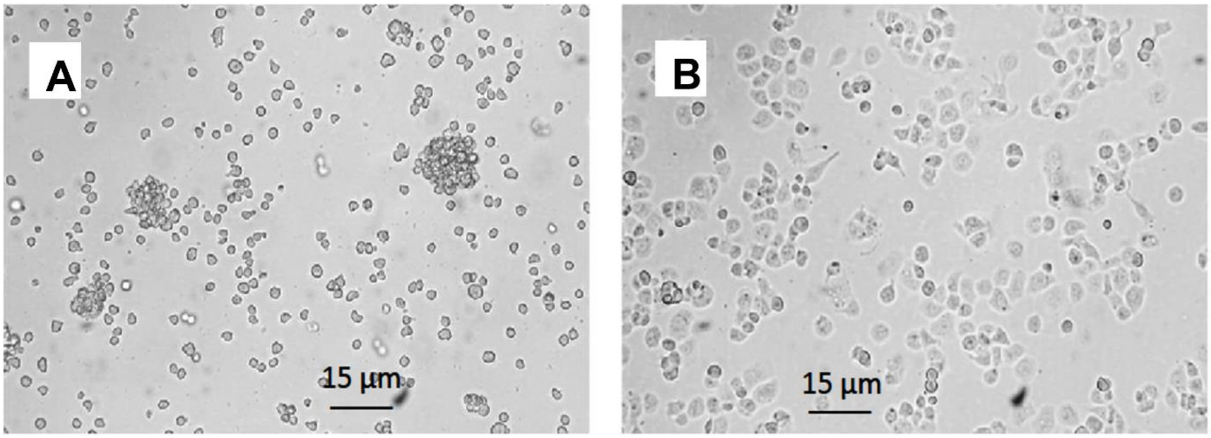

Figure S1. Differentiated THP-1 macrophages display different phenotypes compared to undifferentiated THP-1 monocytes. Representative images of THP-1 cells showing morphological differences of (A) undifferentiated THP-1 cells in culture and (B) THP-1 cells treated with 16 nM (10 ng/mL) PMA for 48 h and allowed to subsequently rest for 24 h. Scale bar = 15 µm.

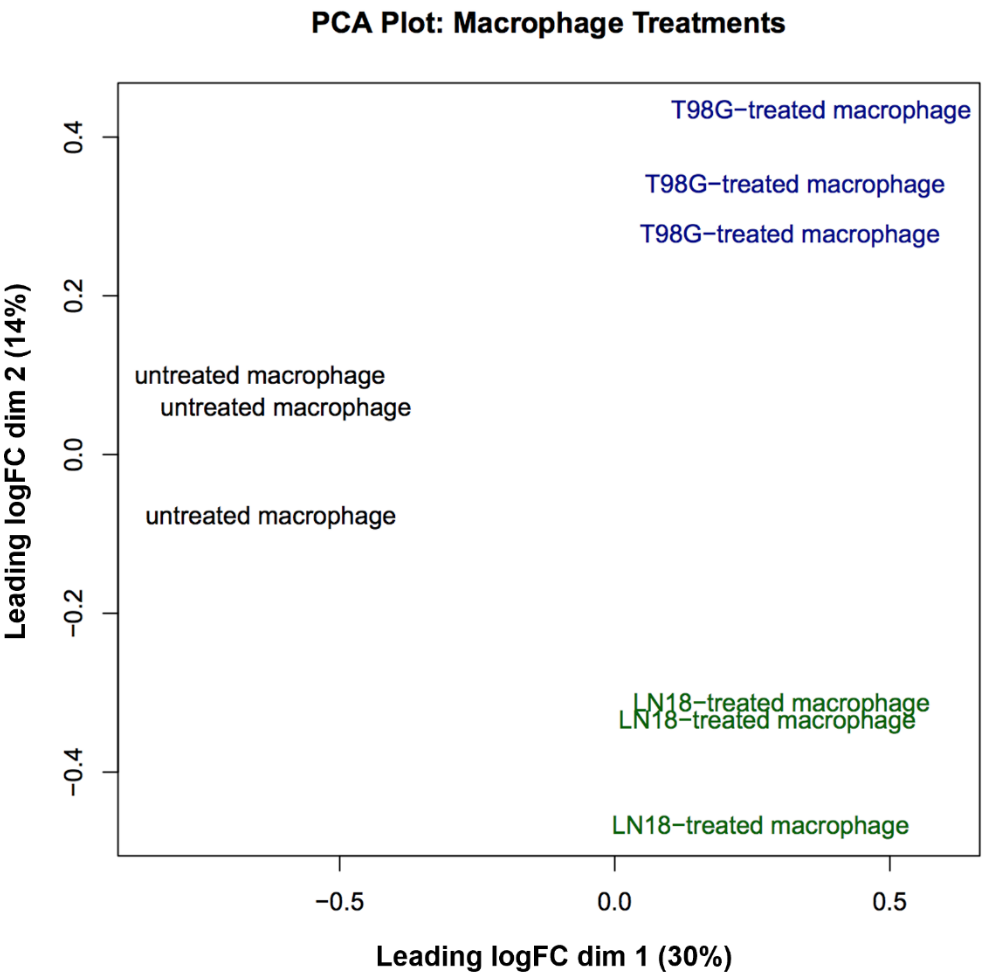

Figure S2. Principle Component Analysis (PCA) plot shows close clustering of all sample replicates, indicating low technical variation.

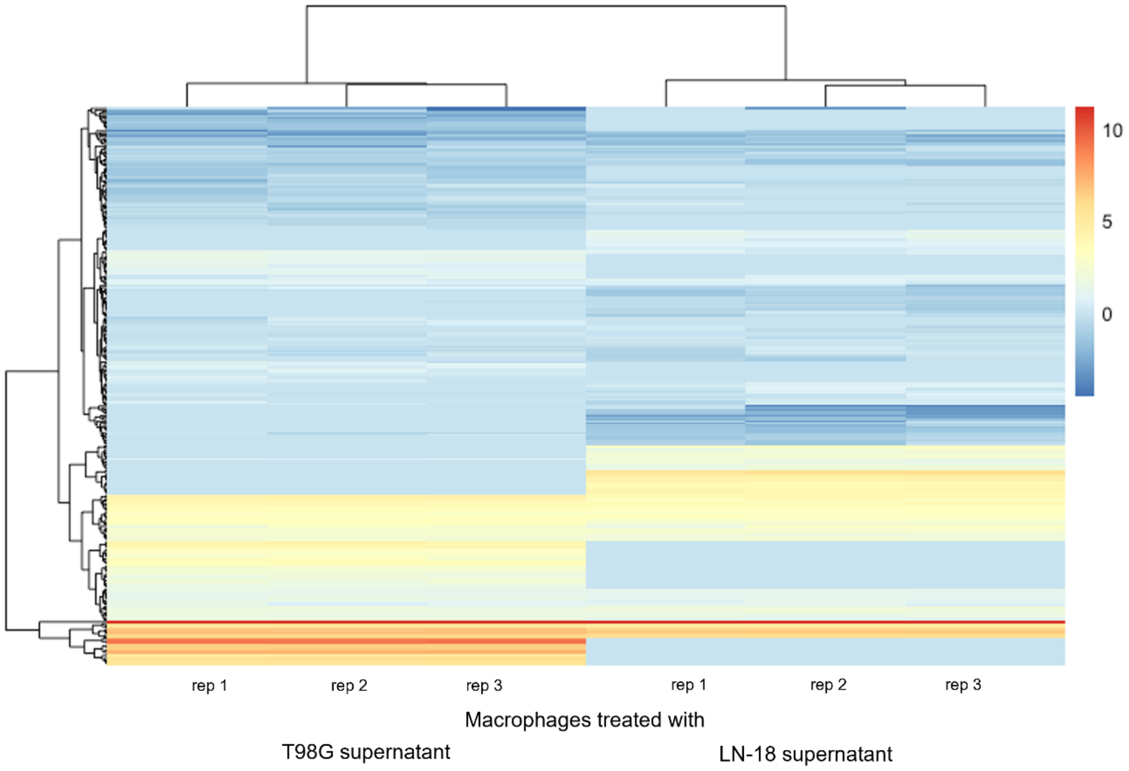

Figure S3. Heatmap of log Fold Change (logFC) of all genes (both in common and not in common) between THP-1 macrophages treated with either supernatant exposed to LN-18 or T98G glioma cells, showing a clear difference in the effect the glioma supernatants have on TPH-1 macrophages. Replicates (rep) refer to biological replicates.
